# A New Treatment-integrated Prognostic Nomogram of the Barcelona Clinic Liver Cancer System for Hepatocellular Carcinoma

**DOI:** 10.1038/s41598-017-08382-3

**Published:** 2017-08-11

**Authors:** Chia-Yang Hsu, Po-Hong Liu, Cheng-Yuan Hsia, Yun-Hsuan Lee, Teddy S. Nagaria, Rheun-Chuan Lee, Shu-Yein Ho, Ming-Chih Hou, Teh-Ia Huo

**Affiliations:** 1 Department of Medicine, Taipei Veterans General Hospital, Taipei: No 201, Sec. 2, Shipai Rd, Taipei, 112 Taiwan; 2 Department of Surgery, Taipei Veterans General Hospital, Taipei: No 201, Sec. 2, Shipai Rd, Taipei, 112 Taiwan; 3 Department of Radiology, Taipei Veterans General Hospital, Taipei: No 201, Sec. 2, Shipai Rd, Taipei, Taiwan; 4 Faculty of Medicine, National Yang-Ming University School of Medicine, Taipei: No 155, Sec. 2, Linong St, Taipei, 112 Taiwan; 5 Institute of Pharmacology, National Yang-Ming University School of Medicine, Taipei: No 155, Sec. 2, Linong St, Taipei, 112 Taiwan; 6 Department of Internal Medicine, University of Nevada School of Medicine Reno, 1155 Mill Street, Reno, NV 89502 USA; 7 Harvard T.H. Chan School of Public Health, Boston: 677 Huntington Ave, Boston, MA 02115 USA; 8 Department of Laboratory Medicine and Pathobiology, University of Toronto, Medical Sciences Building, 6th Floor, 1 King’s College Cir, Toronto, Canada

## Abstract

The nomogram of the Barcelona Clinic Liver Cancer (BCLC) has accurate outcome prediction. This study aims to propose a treatment-integrated nomogram derived from BCLC for patients with hepatocellular carcinoma (HCC). A total of 3,371 patients were randomly grouped into derivation (n = 2,247) and validation (n = 1,124) sets. Multivariate Cox proportional hazards model was used to generate the nomogram from tumor burden, cirrhosis, performance status (PS) and primary anti-cancer treatments. Concordance indices and calibration plots were used to evaluate the performance of nomogram. The derivation and validation sets had the same concordance index of 0.774 (95% confidence intervals: 0.717–0.826 and 0.656–0.874, respectively). In calibration plots, survival distributions predicted by the nomogram and observed by the Kaplan-Meier method were similar at 3- and 5-year for patients from derivation and validation sets. Validation group patients divided into 10 subgroups by the original and new treatment-integrated BCLC nomogram were used to evaluate the prognostic performance of integrating primary anti-cancer treatments. Compared to the nomogram of original BCLC system, the treatment-integrated nomogram of BCLC system had larger linear trend and likelihood ratio X^2^. In conclusion, based on the results of concordance index tests, integrating primary anti-cancer treatments into the BCLC system provides similar discriminatory ability.

## Introduction

Hepatocellular carcinoma (HCC) is the most common primary liver cancer. The American Association for the Study of Liver Diseases and the European Association for the Study of the Liver recommend and endorse the Barcelona Clinic Liver Cancer (BCLC) staging algorithm to be the primary prognostic model and also the allocating tool of primary anti-cancer treatment^1, 2^. We have recently proposed a novel nomogram derived from the BCLC system to provide individualized prognostic prediction for HCC patients^3^. External validation was conducted by a study group from France and showed that the nomogram of BCLC system had better prognostic accuracy compared to the original BCLC system^4^. These data suggest that the nomogram is a reliable and easy-to-use tool for patient cohort with hepatitis B-related HCC and hepatitis C/alcohol-related HCC as well.

Primary anti-cancer treatments are closely associated with the long-term prognosis in HCC. Patients classified within the same BCLC stage could have different primary treatments according to the severity of cirrhosis, tumor burden, and performance status (PS). Curative treatments including surgical resection, transplantation and ablation are recommended by the BCLC algorithm for HCC patients with early stages; however, in order to improve the quality of life or overall survival, a substantial proportion of patients with intermediate to advanced HCC who are at risk for aggressive treatments may also undergo curative therapies^5–7^. Even without strong evidence from large randomized trials, there are abundant studies from around the world showing that more aggressive treatments may provide better clinical outcomes in selected patients^8, 9^.

Although it is well known that treatment modality plays a critical role for HCC patients, no studies to date have questioned if it should be included in the initial cancer staging. This study aims to investigate if primary treatments are associated with long-term prognosis in unselected HCC patients. In addition, a new treatment-integrated nomogram of the BCLC system is proposed and compared with the nomogram of original BCLC system to investigate the prognostic power of incorporating primary anti-cancer therapy into the BCLC system.

## Patients and Methods

### Patients

Between 2002 and 2015, 3,371 newly diagnosed HCC patients in our hospital were prospectively and continuously collected regardless of cancer staging and primary anti-cancer treatment (unselected patient cohort). Collected patients were divided into derivation set (n = 2,247) or validation set (n = 1,124) randomly in this study. The derivation set was used to generate the nomogram model, and the validation set was used to evaluate the accuracy of this nomogram model and the prognostic power of integrating treatments into the BCLC system. The survival status of all patients was checked at 3 months or later after enrollment, and was confirmed by using the database of National Cancer Registry, Taiwan. This study has been approved by the institutional review board (IRB) of Taipei Veterans General Hospital and complies with the standards of the Declaration of Helsinki and current ethical guidelines. Prior to analysis, waiver of consent form from each patient was obtained as justified by the IRB, and patient information was blinded and de-identified.

### Diagnosis and definitions

Findings of typical radiological features in at least two imaging examinations including ultrasound, magnetic resonance imaging (MRI), contrast-enhanced dynamic computed tomography (CT) and hepatic arterial angiography, or by a single positive imaging technique associated with serum α-fetoprotein ≥400 ng/mL or histological confirmation were used to diagnose HCC^10^. Patients with daily consumption of at least 40 g of alcohol for 5 years or more were recorded as alcoholism related^11^. Total tumor volume was calculated based on tumor diameter^12^. Vascular invasion was confirmed by the presence of thrombus adjacent to the tumor in portal system by at least two imaging modalities. Patients received routine chest CT scan to detect metastatic lesion(s) and lymph node involvement. Bone metastasis of HCC was surveyed by bone scan and confirmed by MRI if indicated. The Eastern Cooperative Oncology Group (ECOG) criteria were used to evaluate the overall physical condition of each patient^13^. All clinical data were collected at the time of diagnosis (starting point of follow-up period) and patients were scheduled to receive primary treatment in 1–2 weeks after the diagnosis was made. For patients who did not receive primary treatment in time, re-staging of HCC was performed and the starting point of follow-up was recorded as the date of re-evaluation in the analysis.

### Construction of the nomogram

Three major prognostic factors, tumor burden, cirrhosis and PS, from the BCLC system, and primary anti-cancer treatments were introduced into the multivariate Cox proportional hazards model. Patients with a single tumor smaller than 2 cm in size were coded as tumor burden grade 0. Patients with tumor burden beyond grade 0 and within the Milan criteria (one nodule <5 cm, up to 3 nodules <3 cm, no vascular invasion or extrahepatic involvement) were classified as tumor burden grade 1^3, 14^. Patients were recorded as tumor burden grade 3 if lymph node involvement, vascular invasion, or distant metastasis were confirmed at the time of diagnosis. All remaining patients were coded as tumor burden grade 2. Surgical resection, transplantation and ablation were grouped into curative treatments collectively. Four types of therapy, including curative treatments, transarterial chemoembolization (TACE), targeted therapy (sorafenib) and best supportive care (all medical managements except for the five treatments mentioned above), were introduced into the regression model. No neo-adjuvant chemotherapy was administered before anti-cancer treatments. PS and severity of cirrhosis were coded as the original BCLC system recommends. The ratios of calculated beta coefficients (BETA) from the Cox regression model were used to determine the adjusted prognostic effects of these variables in the nomogram.

### Statistics

The chi-squared test was used to compare categorical data and the Mann-Whitney/Kruskal-Wallis ranked sum test were used for continuous variables (two-tailed). Survival distributions were compared by using the Kaplan-Meier method with a log-rank test. Multivariate Cox proportional hazards model was used to generate the BETAs and hazard ratios. Prognostic discrimination of the nomogram model was examined by the concordance index, which provides the probability that for two randomly selected patients, when one patient has an event (death) after the other, this patient has a better outcome prediction as determined by the nomogram^15, 16^. The calibration plot was generated by comparing the survival distribution observed by the Kaplan-Meier method with the means of nomogram-predicted survival after grouping patients into quintiles. The homogeneity (likelihood ratio X^2^, comparing the fitted model to a model with no predictor) and discriminatory ability (linear trend X^2^, comparing the observed and expected survival times between groups at all time points) were used to evaluate the accuracy of different staging systems in this study^17–19^. Patients in our follow-up program who were not confirmed deceased were recorded as censored. P values < 0.05 were considered statistically significant. All statistical analyses were conducted with the SAS 9.4 (SAS Institute Inc., Cary, NC, USA).

## Results

### Characteristics of study patients

The baseline demographics of patients are shown in Table 1. The mean age of enrolled patients was 65 years, and 77% of them were male. Hepatitis B (54%) is the most common etiology of chronic liver disease. Seventy-three percent of patients were classified as CTP class A and 58% of patients had PS 0. Thirty-seven percent of patients had multiple tumors and 45% of patients had a main tumor diameter of 5 cm or larger. Portal vein invasion was found in 25% of patients and 25% of patients had diabetes mellitus; 9% of patients were confirmed with distant metastasis at the time of data collection.

**Table 1:** Comparison of demographics of the derivation and validation sets.

	All patients (n = 3371)	Derivation set (n = 2247)	Validation set (n = 1124)	p value
Age (years; mean ± SD)	65 ± 13	65 ± 13	65 ± 13	0.6444
Male (n, %)	2580 (77)	1718 (76)	862 (77)	0.8804
Liver disease (n, %)				
Hepatitis B	1807 (54)	1198 (53)	609 (54)	0.6346
Hepatitis C	1023 (30)	670 (30)	353 (31)	0.3444
Alcoholism	632 (19)	417 (19)	215 (19)	0.6893
Tumor size ≥ 5 cm (n, %)	1526 (45)	1027 (46)	499 (44)	0.4712
Multiple tumors (n, %)	1245 (37)	817 (36)	428 (38)	0.3297
Metastasis (n, %)	295 (9)	199 (9)	96 (9)	0.76
Total tumor volume (cm^3^, mean ± SD [median])	367 ± 751	363 ± 738	376 ± 777	0.6999
	(47)	(50)	(48)	
Vascular invasion (n, %)	830 (25)	548 (24)	282 (25)	0.8066
α-fetoprotein ≥ 400 ng/mL (n, %)	987 (30)	665 (30)	322 (29)	0.5688
CTP class (n, %)				0.5971
A	2462 (73)	1640 (73)	822 (73)	
B	748 (22)	494 (22)	254 (23)	
C	161 (5)	113 (5)	48 (4)	
Ascites (n, %)	779 (23)	538 (24)	241 (21)	0.1043
Biochemistry (mean ± SD)				
Albumin (g/dL)	3.7 ± 0.6	3.7 ± 0.6	3.6 ± 0.6	0.5303
Bilirubin (mg/dL)	1.5 ± 2.8	1.6 ± 3	1.4 ± 2.3	0.9299
INR of PT	1.1 ± 0.2	1.1 ± 0.2	1.1 ± 0.2	0.6519
Sodium (mmol/L)	138 ± 4	138 ± 4	139 ± 3.9	0.1014
Estimated GFR ≥ 60 mL/min/1.73 m^2^) (n, %)	2466 (73)	1634 (73)	832 (74)	0.4212
Diabetes mellitus (n, %)	850 (25)	578 (26)	272 (24)	0.3368
Performance status (n, %)				0.8805
0	1955 (58)	1301 (58)	654 (58)	
1–2	1076 (32)	718 (32)	358 (32)	
3–4	340 (10)	228 (10)	112 (10)	
Tumor burden (n, %)				0.7753
0	345 (10)	227 (10)	118 (11)	
1	1086 (32)	718 (32)	368 (33)	
2	1014 (30)	689 (31)	325 (29)	
3	926 (27)	613 (27)	313 (28)	
BCLC stage (n, %)				0.7921
0	264 (8)	182 (8)	82 (7)	
A	824 (24)	536 (24)	288 (26)	
B	551 (16)	370 (16)	181 (16)	
C	1331 (39)	891 (40)	440 (39)	
D	401 (12)	268 (12)	133 (12)	
Treatment (n, %)				0.1472
Resection	973 (29)	630 (28)	343 (31)	
Ablation	648 (19)	448 (20)	200 (18)	
Transplantation	11 (0.3)	7 (0.3)	4 (0.4)	
TACE	940 (28)	621 (28)	319 (28)	
Targeted	133 (4)	82 (4)	51 (5)	
Supportive care	666 (20)	459 (20)	207 (18)	

BCLC, Barcelona Clinic Liver Cancer; CTP, Child-Turcotte-Pugh; GFR, glomerular filtration rate; INR, international normalized ratio; PT, prothrombin time; SD, standard deviation; TACE, transarterial chemoembolization.

A total of 8%, 24%, 16%, 39%, and 12% of patients were classified as BCLC stages 0, A, B, C, and D, respectively at the time of diagnosis. Twenty-nine percent of patients received surgical resection, 19%, 0.3%, 28%, 4% and 20% of patients underwent ablation, transplantation, TACE, targeted therapy and supportive care, respectively. Comparisons between the derivation and validation groups showed no significant differences in all cancer-related variables (all p > 0.05).

### Characteristics of study patients stratified by primary anti-cancer treatments

The baseline demographics of patients receiving different anti-cancer treatments are shown in Table 2. Significantly different patient compositions were found for all HCC-related variables except for diabetes mellitus. Patients receiving curative treatments were more likely to have less severe cirrhosis, smaller tumor burden, better PS, and early BCLC stages. Alternatively, patients receiving supportive care were associated with larger tumor burden, more severe cirrhosis, poorer PS and more advanced BCLC stages.

**Table 2:** Comparison of demographics stratified by primary anti-cancer treatments.

	Curative treatments (n = 1632)	TACE (n = 940)	Targeted therapy (n = 133)	Supportive care (n = 666)	p value
Age (years; mean ± SD)	63 ± 13	67 ± 13	62 ± 15	66 ± 14	<0.001
Male (n, %)	1210 (74)	720 (77)	111 (84)	539 (81)	0.001
Liver disease (n, %)					
Hepatitis B	926 (57)	445 (47)	83 (62)	353 (53)	<0.001
Hepatitis C	503 (31)	323 (34)	23 (17)	174 (26)	<0.001
Alcoholism	272 (17)	179 (19)	33 (25)	148 (22)	0.004
Tumor size ≥ 5 cm (n, %)	391 (24)	491 (52)	115 (87)	529 (79)	<0.001
Multiple tumors (n, %)	404 (25)	460 (49)	54 (41)	327 (49)	<0.001
Metastasis (n, %)	31 (2)	50 (5)	38 (29)	176 (26)	<0.001
Total tumor volume (cm^3^, mean ± SD [median])	144 ± 433	375 ± 768	998 ± 1085	781 ± 980	<0.001
	(14)	(78)	(697)	(524)	
Vascular invasion (n, %)	113 (7)	175 (19)	106 (80)	433 (65)	<0.001
α-fetoprotein ≥ 400 ng/mL (n, %)	281 (17)	256 (27)	90 (68)	360 (54)	<0.001
CTP class (n, %)					<0.001
A	1420 (87)	742 (79)	68 (51)	232 (35)	
B	186 (11)	177 (19)	60 (45)	325 (49)	
C	26 (2)	21 (2)	5 (4)	109 (16)	
Ascites (n, %)	171 (11)	179 (19)	63 (47)	366 (55)	<0.001
Biochemistry (mean ± SD)					
Albumin (g/dL)	3.8 ± 0.6	3.7 ± 0.6	3.5 ± 0.6	3.2 ± 0.6	<0.001
Bilirubin (mg/dL)	1 ± 1	1.1 ± 1	1.9 ± 2.9	3.4 ± 5.4	<0.001
INR of PT	1.1 ± 0.1	1.1 ± 0.1	1.1 ± 0.2	1.2 ± 0.3	<0.001
Sodium (mmol/L)	140 ± 3	140 ± 4	136 ± 4	135 ± 5	<0.001
Estimated GFR ≥ 60 mL/min/1.73 m^2^) (n, %)	1267 (78)	678 (72)	105 (79)	416 (63)	<0.001
Diabetes mellitus (n, %)	395 (24)	242 (26)	38 (29)	175 (26)	0.528
Performance status (n, %)					<0.001
0	1221 (75)	562 (60)	29 (22)	143 (22)	
1–2	374 (23)	330 (35)	87 (65)	285 (43)	
3–4	37 (2)	48 (5)	17 (13)	238 (36)	
Tumor burden (n, %)					<0.001
0	290 (18)	39 (4)	0	16 (2)	
1	799 (49)	240 (26)	2 (2)	45 (7)	
2	410 (25)	457 (49)	21 (16)	126 (19)	
3	133 (8)	204 (22)	110 (83)	479 (72)	
BCLC stage (n, %)					<0.001
0	235 (14)	28 (3)	0	1 (0.2)	
A	636 (39)	168 (18)	1 (1)	19 (3)	
B	263 (16)	243 (26)	7 (5)	38 (6)	
C	443 (27)	443 (48)	107 (81)	338 (51)	
D	55 (3)	58 (6)	18 (14)	270 (41)	

BCLC, Barcelona Clinic Liver Cancer; CTP, Child-Turcotte-Pugh; Curative treatments include resection, ablation and liver transplantation; GFR, glomerular filtration rate; INR, international normalized ratio; PT, prothrombin time; SD, standard deviation; TACE, transarterial chemoembolization.

### Survival analysis of all patients by the BCLC system

**Figure 1:**
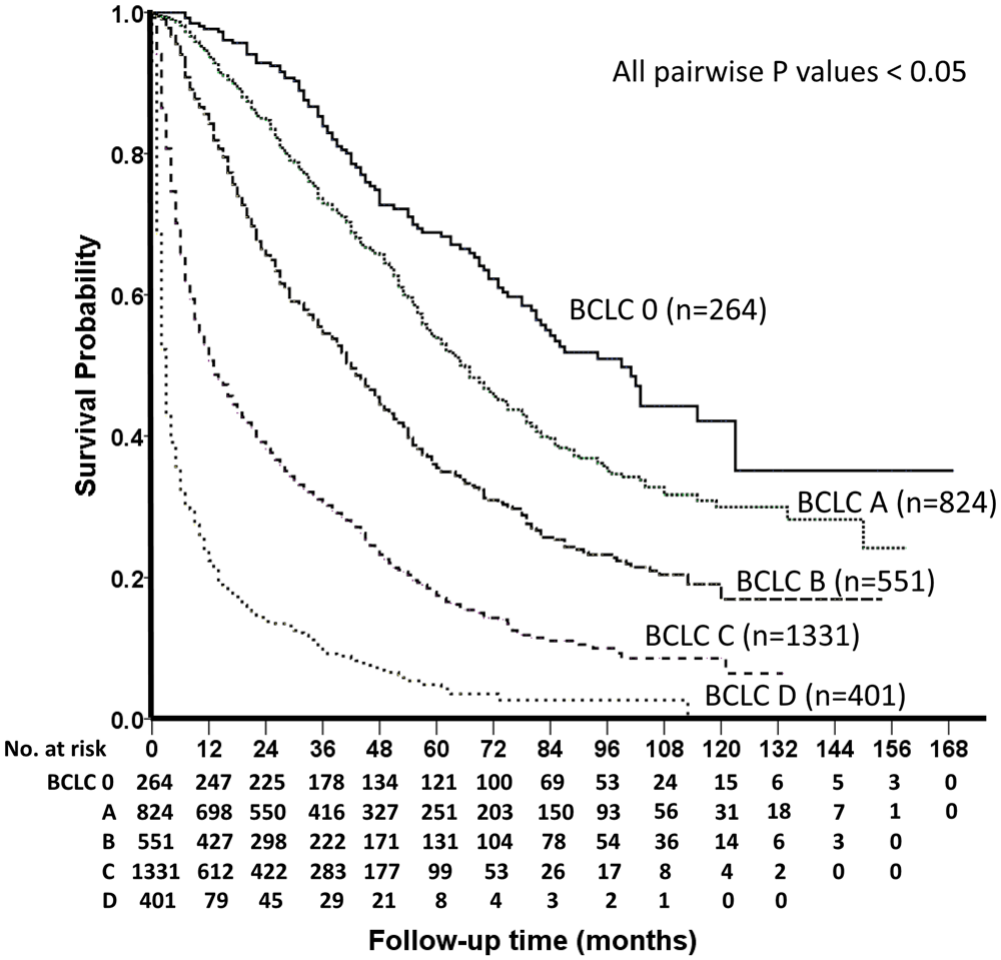
Survival distribution according to the BCLC system. The outcome of patients with early BCLC stage is significantly better than the survival of patients with advanced HCC. All p values for pairwise comparisons are ≤0.05.

With an average follow-up period of 31 (median = 20; range = 1–169) months in this 8,729 person-years study, 64% of patients died. As shown in Fig. 1, patients with more advanced BCLC stages had a significantly decreased survival (all pairwise p < 0.05).

### Survival analysis in Cox regression model of patients from derivation set

The derivation set from randomization was used to determine the prognostic effects of four enrolled BCLC-derived parameters by using the multivariate Cox regression model (Table 3). With PS 0, CTP class A, tumor burden grade 0 and curative treatments as baselines, prognostic effects were calculated for PS 3-4 (BETA = 0.575, p < 0.001), PS 1-2 (BETA = 0.391, p < 0.001), CTP class C (BETA = 0.928, p < 0.001), CTP class B (BETA = 0.464, p < 0.001), tumor burden grade 3 (BETA = 1.383, p < 0.001), grade 2 (BETA = 0.466, p < 0.001), grade 1 (BETA = 0.317, p = 0.007), supportive care (BETA = 1.42, p < 0.001), targeted therapy (BETA = 1.077, p < 0.001), TACE (BETA = 0.689, p < 0.001), respectively.

**Table 3:** Multivariate survival analyses of patients in the derivation set.

	BETA	Nomogram point (BETA*10/BETA of supportive care)	P	Hazard ratio (HR)	95% confidence interval of HR
Performance status 3–4	0.575	4.046	<0.001	1.776	1.46–2.161
Performance status 1–2	0.391	2.751	<0.001	1.478	1.3–1.68
Performance status 0	0			1	
CTP class C	0.928	6.537	<0.001	2.53	1.98–3.233
CTP class B	0.464	3.266	<0.001	1.59	1.387–1.822
CTP class A	0			1	
Tumor burden grade 3	1.383	9.738	<0.001	3.986	3.145–5.051
Tumor burden grade 2	0.466	3.278	<0.001	1.593	1.266–2.004
Tumor burden grade 1	0.317	2.230	0.007	1.373	1.093–1.724
Tumor burden grade 0	0			1	
Supportive care	1.42	10	<0.001	4.137	3.476–4.923
Targeted therapy	1.077	7.583	<0.001	2.935	2.221–3.879
TACE	0.689	4.850	<0.001	1.991	1.744–2.274
Curative treatments	0			1	

BETA, beta coefficient; Curative treatments includes surgical resection, ablation and transplantation.

### Construction of the nomogram

**Figure 2:**
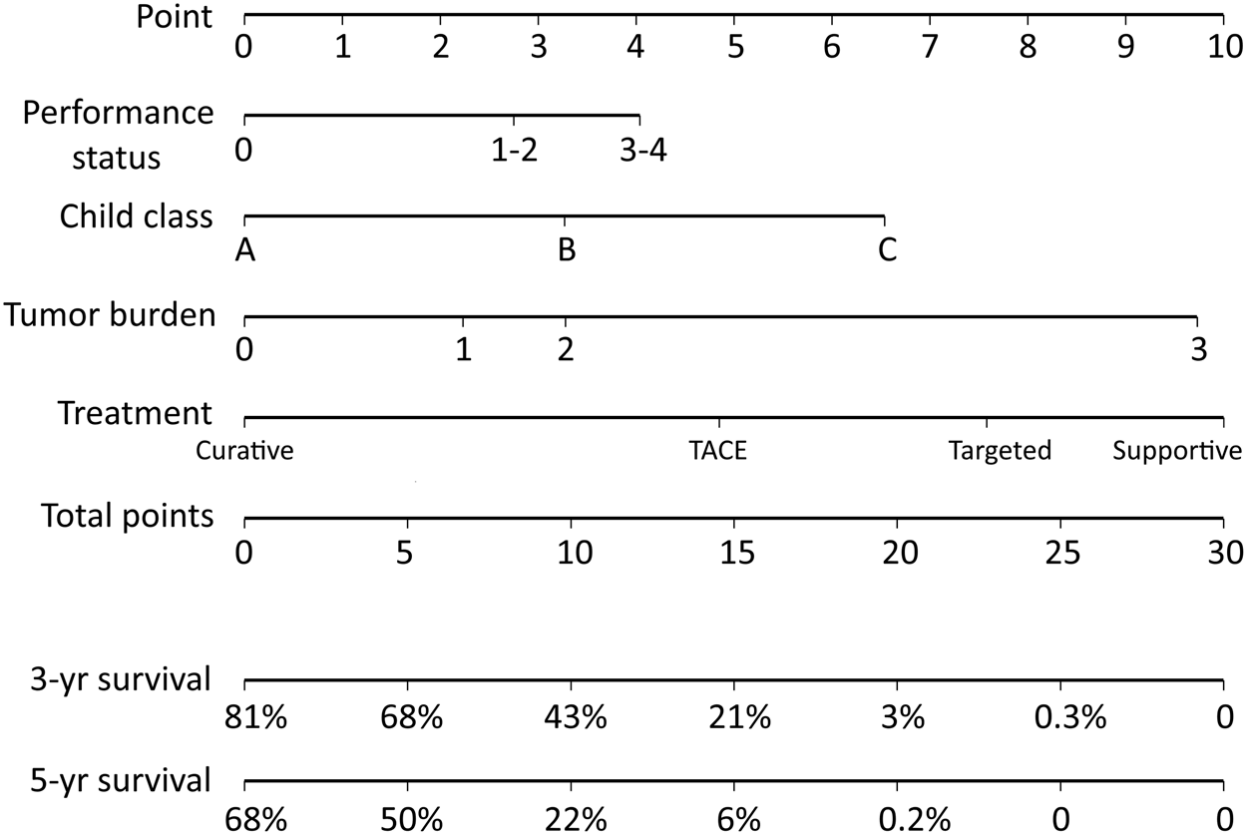
Nomogram predicting 3- and 5-year survival of HCC patients in the derivation set. The nomogram is used by adding up the points identified on the scales of these 4 parameters. The total nomogram point of each patient can be used to predict the probability of survival at 3 and 5 years.

**Figure 3:**
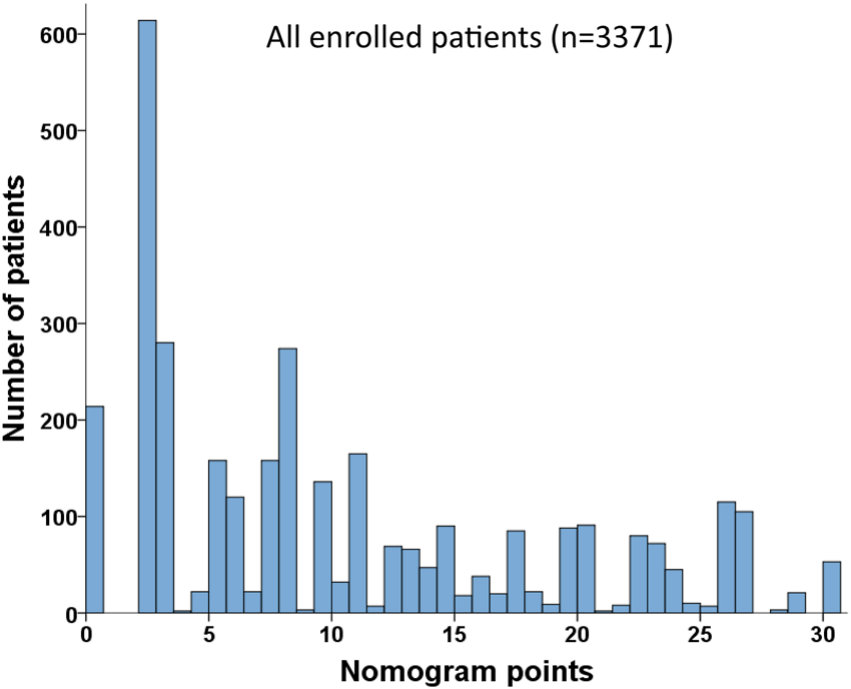
The histogram of nomogram point of all patients. The histogram is right skewed and the range is 0–30.

Supportive care had the highest BETA value in the model and was set as 10 points (Table 3). Sequentially, by using the ratios of BETAs between other prognostic factors and supportive care, 7.6 (calculated as 1.077 divided by 1.42 and timed 10), 4.9, 9.7, 3.3, 2.2, 4.0, 2.8, 6.5, 3.3, points were assigned to targeted therapy, TACE, tumor burden grade 3, grade 2, grade 1, PS 3-4, PS 1-2, CTP class C and class B, respectively. Each patient had one individualized score from 0 to 30 by adding up the points from these 4 prognostic parameters. As shown in Fig. 2, the projections from total points on the scales below indicate the estimated survival probability at 3 and 5 years. The histogram of nomogram score for all patient is illustrated in Fig. 3. Thirty-three percent of patients had a nomogram score less than 5, and 56% of patients had a score less than 10. There were 14.7%, 10%, 10.6% and 9% of patients having a nomogram score between 10–15, 15–20, 20–25 and more than 25, respectively.

### Discrimination and calibration of nomogram in the derivation set

**Figure 4:**
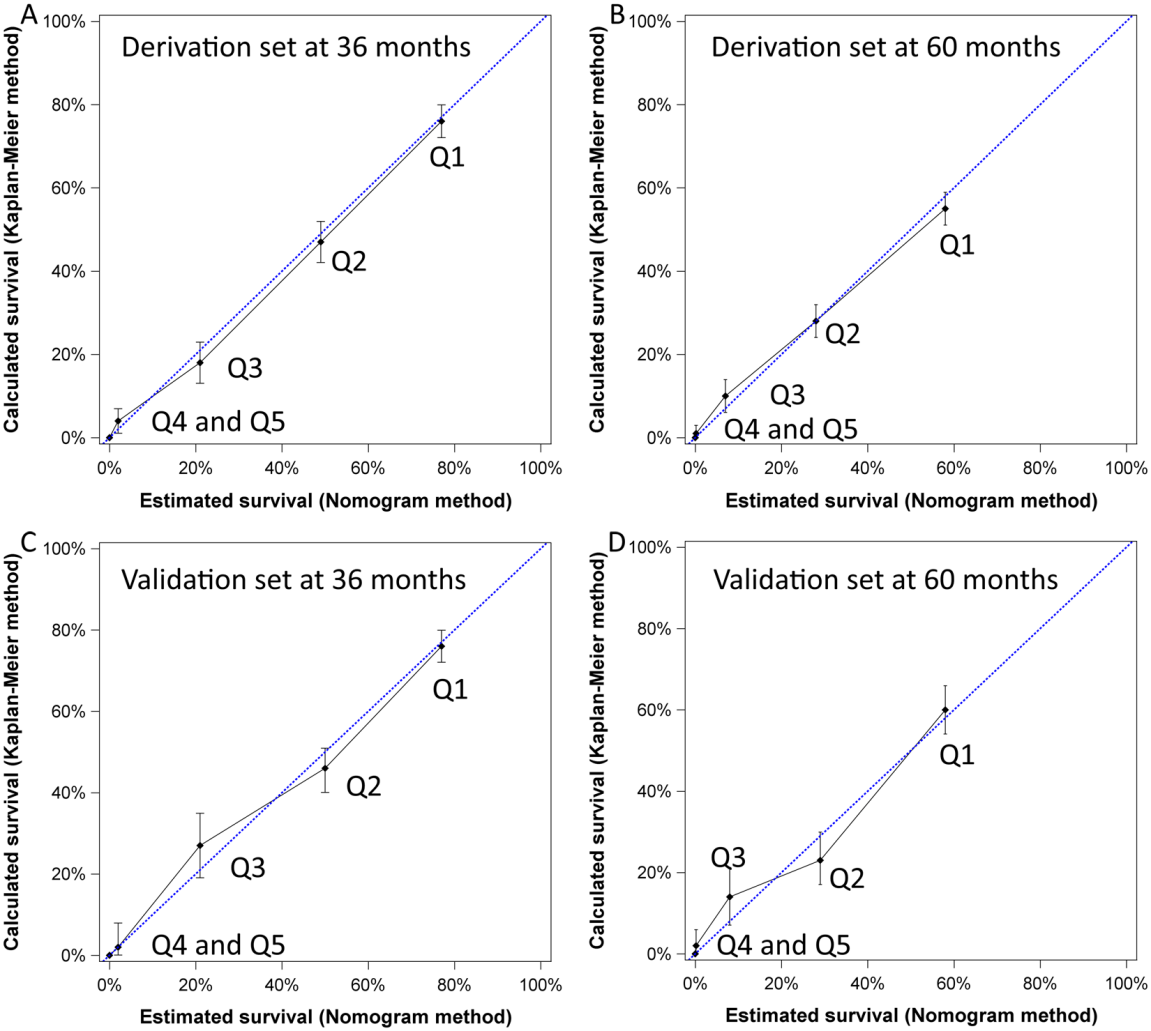
The calibration plots of the nomogram of derivation and validation sets for 3- and 5-year survival predictions. The X-axis represents the nomogram-predicted survival and the Y-axis shows the mean survival and 95% confidence interval calculated by the Kaplan-Meier method. Patients were divided into quintiles (Q) to evaluate the accuracy of nomogram (Q1: ≤6, Q2: 6.1 to 12, Q3: 12.1 to 18, Q4: 18.1 to 24, Q5: >24). For patients from both derivation and validation sets, the calibration lines fit along with the 45-degree reference for both 3- and 5-year survival predictions except for patients nomogram point more than 24.

The nomogram generated from the derivation group had a concordance index of 0.774 (95% confidence interval [CI]: 0.717–0.826). Patients were divided into quintiles (Q) by their nomogram point (Q1: ≤6, Q2: 6.1 to 12, Q3: 12.1 to 18, Q4: 18.1 to 24, Q5: >24) for the calibration plots (Fig. 4). The mean and 95% CI of survival rates calculated by the Kaplan-Meier method are shown on the Y-axis and the mean predicted survival estimated by the nomogram method is shown on the X-axis. The calibration plots for both 3- and 5-year survival well matched the 45-degree line for patients across Q1 to Q4; however, for patients grouped into Q5, the predicted survival were close to 0, which could not be accurately evaluated.

### Discrimination and calibration of nomogram in the validation set

For the validation set, the nomogram had a concordance index of 0.774 (95% CI: 0.656–0.874). As shown in Fig. 4, the calibration plots for 3- and 5-year survival consistently matched the ideal 45-degree reference for patients across all quintiles except for patients grouped into Q5.

### Comparison of the prognostic accuracy between the nomogram of original BCLC system and the treatment-integrated nomogram of BCLC system

Patients from the validation group were used to compare these two nomogram models. The c-index of the nomogram of original BCLC system calculated from validation group was 0.773 (95% CI: 0.653–0.872, nomogram scores: 0–26; Fig. 5). Patients in the validation set were grouped into 10 subgroups by dividing their nomogram scores by 2.6. Similarly, another 10 subgroups were created by dividing their treatment-integrated nomogram point by 3. This 10-level group variable was investigated respectively by the survival analysis equation for both nomogram models; the treatment-integrated nomogram of BCLC system had larger linear trend X^2^ and likelihood ratio X^2^ (Fig. 6).

**Figure 5:**
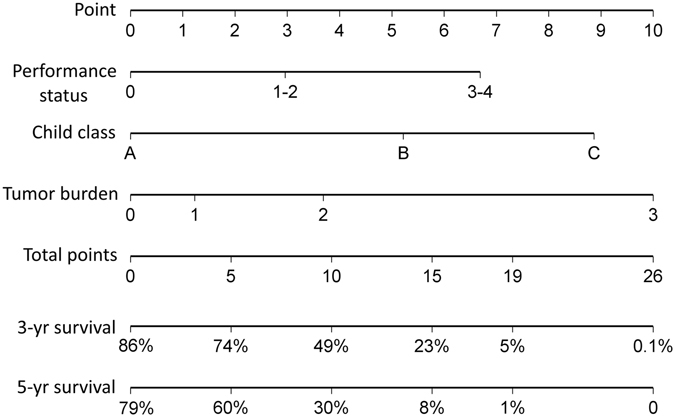
The nomogram of original BCLC system from ref. 3. Three parameters, tumor burden, cirrhosis and PS are used in the nomogram to generate nomogram point between 0–26.

**Figure 6:**
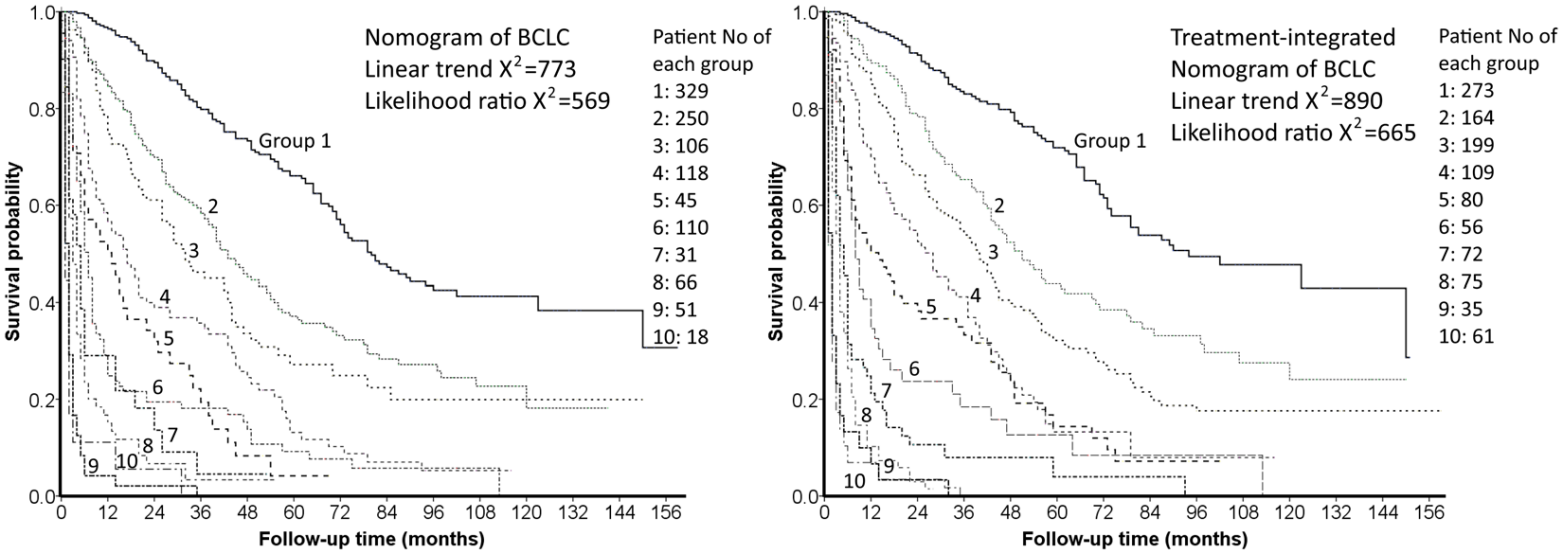
Patients from validation set were divided into 10 subgroups by one-tenth of total nomogram point (2.6 and 3 for the nomogram of original BCLC and the treatment-integrated nomogram, respectively). Compared to the nomogram of original BCLC system, the treatment-integrated nomogram has larger linear trend X^2^ and likelihood ratio X^2^.

## Discussion

For more than a decade, the BCLC system has been recommended by both the US and Europe liver societies as the primary staging system for HCC because it provides accurate survival prediction and treatment allocation guidelines^1, 2^. However, the treatment algorithm is not necessarily strictly followed in different countries; some patients had individualized treatment strategy with the hope to prolong their survival^6, 20^. This study displays that primary anti-cancer treatments are significantly associated with survival after tumor burden, cirrhosis and PS were controlled in the multivariate Cox regression model. This finding prompted us to include primary treatments into the prognostic model. With larger linear trend X^2^ and likelihood ratio X^2^, and similar concordance index, our new nomogram provides similar discriminatory ability when treatments are included in the prognostic model.

The treatment-integrated nomogram has 4 variables, including tumor burden, severity of cirrhosis, PS and treatment. For CTP and PS classification, we used exactly the same design in the BCLC system. Tumor burden grade 0 and 3 were defined by the cutoff provided in the original BCLC system. For the remaining patients, the Milan criteria were introduced to divide patients into tumor burden grade 1 and 2^14^. Surgical resection, transplantation and ablation were grouped into the curative treatments. This user-friendly treatment-integrated nomogram theoretically generates 144 (4 × 4 × 3 × 3) different nomogram scores between 0–30 points for HCC patients. Therefore clinicians may easily calculate the score and predict individualized 3- and 5-year survival for HCC patients with heterogeneous baseline characters.

The concordance indices of both derivation and validations sets were 0.774 in this study. This suggests that if two HCC patients with different nomogram scores are selected, the probability that the patient with a smaller nomogram score lives longer is 77%. Similar results were obtained by 10 times of cross-validation with the same 2:1 splitting to exclude the over-fitting problem. Calibration plots showed consistent results from the nomogram prediction and the survival distribution calculated by the Kaplan-Meier method except for patients with nomogram point higher than 24, which might be related to the fact that most HCC patients with high nomogram points had a very short survival. Altogether, our findings indicate that this treatment-integrated nomogram is a reliable tool to predict individual HCC patient’s 3- and 5-year outcome.

In the Cox regression model, supportive care had the highest BETA value and was given 10 points in the nomogram. Meanwhile, targeted therapy had a nomogram point of 7.6, which was the third strongest predictor in the model. This feature shows the significant association between the overall survival and primary treatments. Notably, after adding primary treatments into the nomogram model, all BETAs of tumor burden, cirrhosis and PS decreased (Figs 2 and 5). Among them, the point of PS 3–4 dropped from 6.6 to 4, which was an apparent difference between these two nomograms. These results could be explained by the strong association between PS and the primary treatments^13, 21^. Integrating treatments into the nomogram may have clinical advantages in providing more detailed prognostic information. Having primary treatments in the nomogram of BCLC system may provide additional 112 (144 vs. 36 [4 × 3 × 3]) possible sets of nomogram scores at most for HCC patients compared to the original BCLC nomogram. Our histogram shows there were approximate 10% of patients with nomogram scores between 15–20, 20–25 and more than 25 each. This finding indicates that our study includes adequate patient number from early to advanced stages, and makes the survival analysis more robust.

For an individual patient, selection of primary treatment could reflect the prognostic effect of some hidden clinical features other than tumor burden, cirrhosis and PS, and this could be an important reason to include primary treatments in the nomogram model. For instance, BCLC stage B patients with chronic renal insufficiency receiving ablation therapy could avoid post-TACE acute renal failure which was often associated with a poor outcome^22^. Recently, multiple propensity score studies showed that selected HCC patients may have a better long-term survival by choosing more aggressive treatments after other cancer-related variables were controlled; their results also suggest that individualized treatment strategy should be included in prognostic models^23–25^. As shown in Fig. 6, compared to the original nomogram, integrating primary treatments improves the likelihood ratio X^2^ (homogeneity) and linear trend X^2^ (monotonicity of gradients), suggesting patients with similar nomogram point had more consistent survival distribution and patients with larger nomogram point had shorter overall survival. In short, integrating primary treatments may improve the prognostic performance of BCLC system, and this easy-to-use, treatment-integrated nomogram could be a better predictive tool for individual patient’s outcome.

This study has some potential limitations. First, curative treatments (surgical resection, transplantation and ablation) may have different effects on survival distribution to some extent; further studies are necessary to address this issue^26–29^. Second, only a very small proportion (11 patients) of patients in our cohort received transplantation as the primary treatment, therefore this nomogram model may not be feasible in medical systems with a high volume of transplantation^30^. Third, this nomogram was built according to the proportional prognostic effects between included variables; if a new treatment is introduced into the guidelines in the future, the predictive accuracy of this nomogram could be reduced. Also, a small proportion (<1%) of patients who did not receive their anti-cancer treatment in 2 weeks after diagnosis might bias the survival analysis when their baseline characteristics were re-evaluated and updated before treatment. Fourth, the patient cohorts used in these two nomograms of BCLC are not independent; among this study cohort, 3179 patients had been reported in the original nomogram of BCLC study^3^. Lastly, the prognostic effect of subsequent anti-cancer treatment(s) was not investigated in this nomogram model, hence the survival probability of some patients may change from initial prediction after receiving subsequent anti-cancer treatments.

In conclusion, the proposed treatment-integrated nomogram is developed from the original BCLC system and primary treatments. This nomogram model is not only the first clinical study to provide quantitative evidence for the rationale of integrating primary treatments into the BCLC system, but also may provide additional survival information for HCC patients on an individual level.
